# Super-sparse principal component analyses for high-throughput genomic data

**DOI:** 10.1186/1471-2105-11-296

**Published:** 2010-06-02

**Authors:** Donghwan Lee, Woojoo Lee, Youngjo Lee, Yudi Pawitan

**Affiliations:** 1 Department of Statistics, Seoul National University, Seoul, South Korea; 2 Department of Medical Epidemiology and Biostatistics, Karolinska Institutet, Stockholm, Sweden

## Abstract

**Background:**

Principal component analysis (PCA) has gained popularity as a method for the analysis of high-dimensional genomic data. However, it is often difficult to interpret the results because the principal components are linear combinations of all variables, and the coefficients (loadings) are typically nonzero. These nonzero values also reflect poor estimation of the true vector loadings; for example, for gene expression data, biologically we expect only a portion of the genes to be expressed in any tissue, and an even smaller fraction to be involved in a particular process. Sparse PCA methods have recently been introduced for reducing the number of nonzero coefficients, but these existing methods are not satisfactory for high-dimensional data applications because they still give too many nonzero coefficients.

**Results:**

Here we propose a new PCA method that uses two innovations to produce an extremely sparse loading vector: (i) a random-effect model on the loadings that leads to an unbounded penalty at the origin and (ii) shrinkage of the singular values obtained from the singular value decomposition of the data matrix. We develop a stable computing algorithm by modifying nonlinear iterative partial least square (NIPALS) algorithm, and illustrate the method with an analysis of the NCI cancer dataset that contains 21,225 genes.

**Conclusions:**

The new method has better performance than several existing methods, particularly in the estimation of the loading vectors.

## Background

Principal component analysis (PCA) or its equivalent singular-value decomposition (SVD) is widely used for the analysis of high-dimensional data. For such gene expression data with an enormous number of variables, PCA is a useful technique for visualization, analyses and interpretation [1-4].

Lower dimensional views of data made possible, via the PCA, often give a global picture of gene regulation that would reveal more clearly, for example, a group of genes with similar or related molecular functions or cellular states, or samples of similar or connected phenotypes, etc. PCA results might be used for clustering, but bear in mind that PCA is not simply a clustering method, as it has distinct analytical properties and utilities from the clustering methods. Simple interpretation and subsequent usage of PCA results often depends on the ability to identify subsets with nonzero loadings, but this effort is hampered by the fact that the standard PCA yields nonzero loadings on all variables. If the low-dimensional projections are relatively simple, many loadings are not statistically significant, so the nonzero values reflect the high variance of the standard method. In this paper our focus on the PCA methodology is constrained to produce sparse loadings.

Suppose *X *is an *n *× *p *data matrix centered across the columns, where *n *and *p *are the number of observations and the number of variables, respectively. Also, let *S*_*X *_= *X*^*T*^*X/n *be the sample covariance matrix of *X*. In PCA, the interest is to find the linear combination *z*_*k *_= *Xv*_*k*_, for *k *= 1, ..., *p*, which maximizes(1)

with the constraints  and *v*_*k *_⊥ *v*_*h *_for all *h < k. *PCA can be computed through the SVD of *X*. Let the SVD of *X *be(2)

where *D *is *n *× *p *matrix with (*i, i *)th element *d*_*i*_; the columns of *Z *= *UD *= *XV *are the principal component scores, and the columns of the *p *× *p *matrix *V *are the corresponding loadings. The vector *v*_*k *_in (1) is the *k*-th column of *V*.

Each principal component in (2) is a linear combination of *p *variables, where the loadings are typically nonzero so that PCA results are often difficult to interpret. To get sparse loadings, [5] proposed to use *L*_1_-penalty, which corresponds to the least-absolute shrinkage and selection operator (LASSO; [6]). [7] proposed to use the so-called elastic-net (EN) penalty. However, LASSO and EN may not be satisfactory either, because it can still gives too many nonzero coefficients. [8] proposed the smoothly-clipped absolute deviation (SCAD) penalty for oracle variable selection. Recently, in regression setting, [9] proposed a new random-effect model using a gamma scale mixture, which gives various types of penalty, including the normal-type (bell-shaped for ridge penalty), cusped-type (LASSO and SCAD-type), and a new (singular) unbounded penalty at the origin. [9] showed that the new unbounded penalty can yield very sparse estimates that are better than LASSO both in prediction and sparsity.

In this paper we use the random-effect model approach of [9] for sparse PCA (SPCA); the model gives unbounded gains for zero loadings at the origin, so it forces many estimated coefficients to zero. We improve the estimation further by shrinking the singular values from the SVD of the data; the resulting procedure is called super-sparse PCA (SSPCA). We provide some simulation studies that indicate that these SPCA methods perform better than existing ones, and illustrate their use using a cancer gene-expression dataset with 21,225 genes. We also show how to modify the ordinary NIPALS algorithm [10] to implement these methods computationally.

## Results

### Numerical studies

We first perform small simulation studies in order to assess the performance of the proposed sparse PCA methods and compare them against other methods. We generate data matrix *X *= (*X*_1_*,…,X_p_) *where *X*_*i *_∈ *R*^*n*^, as follows:(3)(4)

where , *e*_*i *_~ *MVN*(0, *ϕI*_*n*_), *I*_*n *_is the identity matrix of order *n *and *u *and *e*_*i *_are independent for all *i. *This gives the true covariance matrix,

where , Σ_22 _= *ϕI*_*p*-4 _and *J_k _*is the *k × k *matrix of ones. Here we consider cases (*n*, *p*) = (80, 20) for *n *>*p *and (*n*, *p*) = (50, 200) for *n *<*p*. Based on 100 simulated data, we compare our new sparse PCA method using the h-likelihood (HL; See Methodology section) with the LASSO and EN penalties for both SPCA and SSPCA methods. We also tried the SCAD method but the results are very similar to LASSO, so we do not report results for SCAD.

From the SVD of Σ we have the true first loading vector *v*_1 _= (1/2,1/2,1/2,1/2,0, ..., 0)^*T*^. Let  be the estimate of *v*_1_. To evaluate the performance in estimation of the first loading vector, following [11], we use the sine values of the angle between true loading and estimated loading as the measure of the closeness of two vectors, i.e.

When *v*_1 _= , dist (*v*_1_, ) = 0.

The summary of estimation performance is given in Table 1. Generally SPCA methods have much better estimation than the ordinary PCA method. Among SPCA methods, the condition-number constrained SSPCA method with HL is generally the best, although the improvement over the unconstrained method is not substantial. The improvement performance of HL over LASSO and EN is small when *n *>*p*, but it is substantial when *n *<*p *and the underlying signal is not very strong ( = 0.5).

**Table 1:** Simulation results: estimation

						SPCA	
*n*	*p*			PCA	HL	LASSO	EN
80	20	2.0	0.1	0.054 (0.010)	0.023 (0.011)	0.022 (0.010)	0.025 (0.013)
		0.5	0.1	0.109 (0.021)	0.045 (0.021)	0.051 (0.022)	0.055 (0.029)
50	200	2.0	0.1	0.223 (0.022)	0.029 (0.014)	0.035 (0.015)	0.056 (0.028)
		0.5	0.1	0.424 (0.041)	0.062 (0.032)	0.080 (0.033)	0.122 (0.058)

				PCA*	**HL**	**SSPCA ** **LASSO**	**EN**

80	20	2.0	0.1	0.055 (0.010)	0.020 (0.009)	0.021 (0.010)	0.022 (0.010)
		0.5	0.1	0.113 (0.020)	0.042 (0.020)	0.050 (0.023)	0.050 (0.026)
50	200	2.0	0.1	0.218 (0.025)	0.026 (0.013)	0.032 (0.014)	0.055 (0.030)
		0.5	0.1	0.993 (0.010)	0.063 (0.030)	0.083 (0.044)	0.866 (0.000)

The median of dist(*v*_1_, ) and the median absolute deviation in parentheses.

PCA*: PCA using X*

To evaluate the performance in variable selection, in Table 2 we report the percentage of selecting the true model (correctly identifying all of the true zero elements), the median number of correctly estimated zeroes divided by the number of true zeroes (true negatives) and incorrect zero estimates divided by the number of true nonzeroes (false negative). Because it does not produce zeroes, the ordinary PCA method never gets the true model and always gets 0 true negatives and 0 false negatives. The HL penalty outperforms the LASSO penalty and generally better than the EN penalty, particularly in identification of the true model. LASSO identifies fewer true negatives compared to HL. The SSPCA methods with the HL and LASSO penalties outperform the corresponding SPCA methods, but here again HL is better than LASSO and EN. The EN performs worst when *n *<*p *and the underlying signal is not very strong ( = 0.5).

**Table 2:** Simulation results: model selection

						SPCA				SSPCA	
*n*	*p*			PCA	HL	LASSO	EN	PCA*	HL	LASSO	EN
80	20	2.0	0.1	0	72	12	64	0	95	14	99
				0/16	16/16	14/16	16/16	0/16	16/16	15/16	16/16
				0/4	0/4	0/4	0/4	0/4	0/4	0/4	0/4

		0.5	0.1	0	77	1	56	0	100	43	99
				0/16	16/16	12/16	16/16	0/16	16/16	15/16	16/16
				0/4	0/4	0/4	0/4	0/4	0/4	0/4	0/4

50	200	2.0	0.1	0	73	0	88	0	100	27	87
				0/196	196/196	184.5/196	196/196	0/196	196/196	194/196	196/196
				0/4	0/4	0/4	0/4	0/4	0/4	0/4	0/4

		0.5	0.1	0	79	0	70	0	97	84	0
				0/196	196/196	185.5/196	196/196	0/196	196/196	196/196	196/196
				0/4	0/4	0/4	0/4	0/4	0/4	0/4	3/4

Percentages of selecting the true model, the median number of correct 0 divided by the number of zeroes and incorrect 0 divided by the number of non-zeroes.

PCA*: PCA using X*

Finally, as a measure of the prediction power, we compute the test sample variance,

where  is the estimated loading using data matrix  and *X*_test _is the independent test data sets generated from (3) with same sample size *n*. The results are in Table 3. SPCA methods give better prediction power than the ordinary PCA. Except for the SSPCA method when *n *<*p *and the underlying signal is not very strong ( = 0.5), six SPCA methods have similar prediction power.

**Table 3:** Simulation results: prediction

						SSPCA	
*n*	*p*			PCA	HL	LASSO	EN
80	20	2.0	0.1	7.979 (0.831)	7.998 (0.837)	7.996 (0.842)	7.970 (1.116)
		0.5	0.1	2.050 (0.213)	2.057 (0.222)	2.055 (0.225)	2.088 (0.283)
50	200	2.0	0.1	7.907 (1.633)	8.242 (1.599)	8.242 (1.601)	8.149 (1.386)
		0.5	0.1	1.769 (0.362)	2.143 (0.418)	2.140 (0.414)	2.071 (0.349)

						**SSPCA**	
				PCA*	**HL**	**LASSO**	**EN**

80	20	2.0	0.1	7.954 (1.125)	7.999 (0.849)	7.997 (0.850)	7.978 (1.115)
		0.5	0.1	2.062 (0.292)	2.057 (0.226)	2.057 (0.225)	2.088 (0.280)
50	200	2.0	0.1	7.564 (1.718)	8.243 (1.593)	8.243 (1.597)	7.928 (1.755)
		0.5	0.1	0.242 (0.075)	2.137 (0.452)	2.149 (0.424)	0.503 (0.316)

The median of test variance with the median absolute deviation in parentheses.

PCA*: PCA using X*

### Analysis of NCI data

In the analysis of microarray data it is often of interest to co-regulated genes, since they will point to some common involvement in molecular functions or biological processes or cellular states. PCA is a useful tool for such analyses [1-4]; since interpretation depends on comparing the relative sizes of the loading vectors, the sparse loadings in SPCA are much easier to interpret than ordinary PCA. Furthermore, the previous section also shows that SPCA has better estimation characteristics than the ordinary PCA. For illustrations we consider the so-called NCI-60 microarray data downloaded from the CellMiner program package, National Cancer Institute http://discover.nci.nih.gov/cellminer/. Only *n *= 59 of the 60 human cancer cell lines were used in the analysis, as one of the cell lines had missing microarray information. The cell lines consist of 9 different cancers and were used by the Developmental Therapeutics Program of the U.S. National Cancer Institute to screen > 100,000 compounds and natural products. The number of genes is *p *= 21,225.

**Figure 1:**
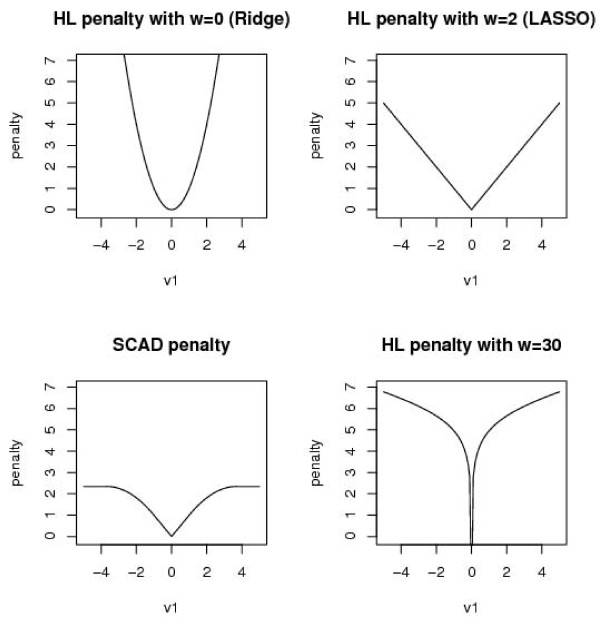
**HL penalty functions associated with the ridge (*w *= 0), LASSO (*w *= 2), SCAD and when *w *= 30**.

Figure 1 in the Additional file 1 gives the plots of the estimates of first loading of 21,225 variables (genes) from five PCA methods, and Table 4 shows the proportion of zero coefficients (< 0.00005) of first loading vector. The ordinary PCA has almost all nonzero loadings (99%); to interpret the results, one must apply a threshold value on the coefficients, but it is not obvious how to choose the threshold. SPCA with LASSO penalty gives only 3% zero loadings, so it is not sparse; HL penalty give more sparse loadings (37.5% zeroes), but the proportion of nonzero loadings are still quite large. SSPCA with LASSO penalty is slightly improves, with 5.4% zeroes, but it is still far from sparsity with more than 20,000 nonzero loadings. Here SSPCA with HL penalty gives the most sparse loadings, with only 6% nonzero loadings, so we have managed to force almost 20,000 loadings to zero.

**Table 4:** Analyses of NCI data: number of zero loadings

PCA	SPCA		SSPCA	
	HL	LASSO	HL	LASSO
214/21225	7966/21225	650/21225	19965/21225	1144/21225
(1.01)	(37.53)	(3.06)	(94.06)	(5.39)

The proportion (percentage) of zero elements of first loading in NCI data analysis.

To select the number of principal components, we use a permutation approach as follows. First, we randomly permute the expression values within each sample (row) of *X *to create permuted data *X*_*perm*_. Then PCA is performed on *X*_*perm *_to get the singular values . We perform *P *= 1000 permutations, from which we can compute the p-values of the observed *d*_*k*_'s. The number of principal components, *k*_0_, is such that the p-value of *d*_*k*_'s is less than 0.001 when *k *≤ *k*_0_.

For NCI data, we get *k*_0 _= 8 (eight significant principal components). The numbers of nonzero elements in the eight loading vectors (*v*_1_…*v*_8_) are given in Table 5. We also report the adjusted variance and cumulative adjusted variance as suggested by [7] to get the explained variance properly when the principal component scores are correlated. Note that the adjusted variance is equal to the variance in the ordinary PCA because the principal components of PCA are uncorrelated. Despite the sparsity, in comparison with the ordinary PCA, both of SPCA and SSPCA method give higher cumulative adjusted variance. In fact the SSPCA method gives extremely sparse results, with only 1,260, 681 and 375 nonzero loadings for the first 3 principal components, compared to 13,259, 4,086 and 15,362 for the SPCA method. Up to the third principal component the latter has only slightly larger cumulative variance.

**Table 5:** Analysis of NCI data: number of zero loadings

Principal component scores	***Z***_**1**_	***Z***_**2**_	***Z***_**3**_	***Z***_**4**_	***Z***_**5**_	***Z***_**6**_	***Z***_**7**_	***Z***_**8**_
PCA								
Number of nonzero loadings	21011	20385	19226	21099	20948	20817	20945	20997
Adjusted Variance (%)	12.3	10.2	6.6	4.1	3.6	3.2	2.9	2.6
Cumulative adjusted Variance (%)	12.3	22.5	29.1	33.2	36.8	40.0	42.9	45.5

SPCA - HL								
Number of nonzero loadings	13259	4086	15362	13547	13946	10445	9890	10958
Adjusted Variance (%)	20.6	13.4	11.5	6.4	6.1	4.9	4.0	4.1
Cumulative adjusted Variance (%)	20.6	34.0	45.5	51.9	58.0	62.9	66.9	71.0

SSPCA - HL								
Number of nonzero loadings	1260	681	375	290	47	58	33	3434
Adjusted Variance (%)	22.3	8.7	6.1	6.5	1.3	0.4	0.0	1.6
Cumulative adjusted Variance (%)	22.3	31.0	37.1	43.6	44.9	45.3	45.3	46.9

Number of nonzero loadings and cumulative variance for different methods.

Figure 2 in the Additional file 1 shows the scatterplot matrix of the first 3 SSPCA scores. Except for breast cancer, the different cancer types appear in recognizable clusters in the plot. This means that the sparse vector loadings capture some underlying biological differences between the cancers. To find biological explanation, Table 6 shows the Gene Ontology (GO) [12] biological processes enrichment analyses of the nonzero loadings from the first 3 principal components from SSPCA. Only the top 20 most enriched categories with P-values < 10^-5 ^are shown. The results indicate that the greatest variation in gene expression are associated with structure development, cell proliferation and cell death (apoptosis), and cell adhesion. These processes are closely related to the hallmarks of cancer progression such as angiogenesis (development of blood vessels), abnormal cell growth and eventually metastasis (cell migration made possible by abnormally low cell adhesion).

**Table 6:** Gene Ontology analysis

Number	GO ID	GO Term	P-value(1)	P-value(2)	P-value(3)
1	GO:0048856	anatomical structure development	1.6e-10	1.5e-09	4.5e-07
2	GO:0009653	anatomical structure morphogenesis	2.9e-10	4.8e-06	
3	GO:0008283	cell proliferation	1.3e-09		
4	GO:0050793	regulation of developmental process	1.7e-09	9.4e-06	
5	GO:0032502	developmental process	3.8e-09	8.1e-08	4.9e-06
6	GO:0042127	regulation of cell proliferation	5.8e-08	3.9e-06	
7	GO:0048513	organ development	6.6e-08		
8	GO:0048869	cellular developmental process	1e-07		
9	GO:0048731	system development	1.1e-07	3.6e-07	5.3e-06
10	GO:0007155	cell adhesion	1.3e-07	7.6e-07	
11	GO:0022610	biological adhesion	1.3e-07	7.6e-07	
12	GO:0051093	negative regulation of developmental process	1.9e-06		
13	GO:0048519	negative regulation of biological process	2.8e-06		
14	GO:0048523	negative regulation of cellular process	3.4e-06		
15	GO:0009605	response to external stimulus		2.8e-07	
16	GO:0043065	positive regulation of apoptosis		7.4e-06	
17	GO:0043068	positive regulation of programmed cell death		8.6e-06	
18	GO:0042981	regulation of apoptosis		9.6e-06	
19	GO:0032501	multicellular organismal process			1.3e-06
20	GO:0007275	multicellular organismal development			4.3e-06

The top 20 most enriched biological process GO terms and the associated P-values for the first three principal components from SSPCA.

Comparative GO analyses from the ordinary PCA are given in the Additional file 1. We use the same number of top-ranking nonzero loadings as for the SSPCA, which are 1,260, 681 and 375 for the first 3 principal components, respectively. Out of these, the number of overlapping probes between the SSPCA and PCA are 462, 194 and 60. These overlaps are substantially more (up to 8 times more) than expected under random re-arrangement. However, there is sufficiently large number of distinct probes in the two methods, so the GO analyses could be different. The P-values from the SSPCA-based GO analyses are more significant than those from the ordinary PCA; this may be due to better estimation of the loadings, so that the SSPCA has better power than the ordinary PCA in revealing biologically-important grouping of genes.

## Discussions and Conclusions

PCA is one of the most important tools in multivariate statistics, where it has been used, for example, in data reduction or visualization of high-dimensional data. The emergence of ultra-high dimensional data such as in genomics, involving 10,000s of variables but with only a few samples has brought new opportunities for PCA applications. However, there are new challenges also, particularly on the interpretation of results. If we treat PCA quantities such as the loading vectors as parameter estimates, the large-*p*-small-*n *applications typically produce very noisy estimates. This is obvious since the loading vectors are a statistic derived from the sample covariance matrix, and the latter is not well estimated.

It is well known that improved estimation can come by imposing constraints, and in this case sparsity constraint is natural. As PCA scores capture some underlying biological processes, we do not expect every gene in the genome to be involved. Out of possibly 30,000 genes we can expect only a small fraction, probably less than 1,000, to be involved in a cellular process. Hence sparsity constraint can help in reducing the number of loading parameters to estimate.

Imposing statistical constraints can be achieved by applying a penalty approach as used by the ridge regression or the LASSO methods [6]. In this paper we have investigated a random-effect model approach using a gamma scale mixture, which leads to a class of penalties that includes the ridge and LASSO penalties as special cases. One significant property is that it can produce unbounded penalties on the origin, which leads to stronger constraints and more sparse estimates. From our results it seems clear that the penalty approach alone is not able to yield sufficiently sparse PCA for high-dimensional genomic data. Additionally we also need the shrinkage on the singular values of the data matrix. In simulation studies we show that the proposed methods outperform existing methods both in estimation and model selection. Hence we believe that the new SPCA methods are promising tools for high-dimensional data analyses.

For future works, it will be of interest to apply super-sparse technique in this paper to locally-linear methods of dimensionality reduction (e.g. [13]]), partial-least squares (PLS) regression and classification methods (e.g. [14]), or other high-throughput data analysis method where dimensionality reduction is used (e.g. [15]).

## Methodology

### NIPALS algorithm for PCA

Standard algorithms for SVD (e.g. [16]) give the PCA loadings, but if *p *is large and we only want to obtain a few singular vectors, the computation to obtain the whole set of singular vectors may be impractical. Furthermore, with these algorithms it is not obvious how to impose sparsity on the loadings. [10] described a NIPALS algorithm that works like a power method ([17], p.523) for obtaining the largest eigenvalue of a matrix and its associated eigenvector. The NIPALS algorithm computes only a singular vector at a time, so it is efficient if we only want to extract a few singular vectors. Also the steps are recognizable in regression terms, so the algorithm is immediately amenable to random-effect modification as needed to obtain the various SPCA methods proposed in this paper.

First we review the ordinary NIPALS algorithm: Set the initial value of *z*_1 _as the first column of *X*, then

1. Find 

2. Normalize 

3. Find *z*_1_: *z*_1 _← *X*^*T*^*v*_1_

4. Repeat steps 1 to 3 until convergence.

To obtain the second-largest singular value, first compute residual , then apply the NIPALS algorithm above by replacing *X *by *X*_2_.

### Sparse PCA via random-effect models

To impose sparseness on the PCA loadings we first introduce the regression framework into step 1 of the NIPALS algorithm. Denoting *X*_*j *_as the *j*th column of *X*, following [18] we have

where *v*_1*j *_is the *j*th element of the *p *× 1 vector *v*_1 _(the first loading vector), and ∊_*j *_is an error term. If *z*_1 _is assumed to be known, the ordinary least square (OLS) estimate for *v*_1 _is given by

Consider the penalized least-squares (PLS) estimation that minimizes(5)

where *p*_*λ*_(·) is a penalty function. For example, *p*_*λ*_(|*v*_1*j*_|) = *λ*|*v*_1*j*_| gives LASSO,  gives ridge, and  gives EN, where *λ*, *λ*_1 _and *λ*_2 _are tuning parameters. For the prediction the ridge-type penalty is effective and for sparse estimation the LASSO-type penalty is recommended, so that EN [19] has been recommended as a compromise between the ridge and LASSO methods. [7] proposed to use EN for sparse (SPCA), but it gives less sparse estimates than LASSO.

[9] recently proposed the use of random-effect models to generate new penalty functions for sparse regression estimation. Suppose that *v*_1*j *_is a random variable such that(6)

where *θ *is the dispersion parameter and *u*_*j *_follows the gamma distribution with a parameter *w *and density

such that E(*u*_*j*_) = 1 and Var(*u*_*j*_) = *w. *This model leads to a rather complex marginal distribution for *v*_1*j, *_characterized by parameter *w *and with density

This model involves a computationally difficult integral, and its direct optimization is problematic due to the nonconvexity of -log *f_w, θ_*(*v*_1*j*_). To overcome these problems, first note that the random-effect model (6) can be written as(7)

where *τ*_*j *_= *u*_*j*_*θ *and *e*_*j *_~ *N*(0,1). This is the double hierarchical generalized linear model [20]. With the log link, we have an additive model

This leads to the h-likelihood (HL) of [21](8)

where

and *f*_*θ*_(*v*_1*j*_|*u*_*j*_) and *f*_*w*_(log *u*_*j*_) are the density functions of *u*_1*j*_|*u*_*j *_and log *u*_*j*_, respectively. Given (*w*, *ϕ*, *θ*), for the estimation of *v*_1_, [9] proposed to use the profile h-likelihood

where  solves *dh*/*du *= 0.

[9] showed that(9)

with , and the estimate of *v*_1 _can be found using the iterative weighted least squares (IWLS) by solving(10)

using  and *λ *= *ϕ*/*θ*. In random-effect model approach, the penalty function *p*_*λ*_(|*v*_1*j*_|) stems from a probabilistic model . As noted previously the proposed penalty *p*_*λ*_(|*v*_1*j*_|) is nonconvex. However, by expressing the model for *p*_*λ*_(|*v*_1*j*_|) hierarchically as (i) *v*_1*j*_|*u*_*j *_is normal and (ii) *u*_*j *_is gamma, both models can be fitted by convex GLM optimizations. Thus, the proposed IWLS algorithm overcomes the difficulties of a nonconvex optimization by solving two-interlinked convex optimizations [22].

Figure 1 shows HL penalties *p*_*λ*_(|*v*_1*j*_|) at *w *= 0, 2, and 30, and SCAD penalty at λ = 1. The form of the penalty changes from a quadratic shape (*w *= 0) for ridge regressions to a cusped form (*w *= 2) for LASSO and then to an unbounded form (*w *> 2) at the origin. In the case of *w >*2, it allows an infinite gain at zero. Bell-shaped penalties have been proposed for better prediction (e.g., [23]), and cusped ones for simultaneous variable selection and estimation as in LASSO [6] or SCAD [24]. Until now, however, only finite penalties have been investigated. [9] proposed to use the unbounded penalty with *w *= 30, which we shall call the HL method. They illustrated the advantage of using this unbounded penalty to enhance sparse coefficient estimation. Singularities in LASSO and SCAD occur as the penalty functions have no derivatives at the origin. However, both penalties have |*p*_*λ*_(0)| < ∞ and , while the new unbounded penalty has |*p*_*λ*_(0)| < ∞ and .

In general, the minimizer of the penalized least-squares (5) can be found using the IWLS (10) with . The derivative  for LASSO, SCAD and HL penalties are summarized in Table 7. When *v*_1*j *_= 0, then  and the *j*th element of *W*_*λ *_is not defined. [9] employed a perturbed random-effect estimate  for a small positive * δ *= 10^-8^. Then,  is always defined. As long as *δ *is small, the diagonal elements of *W*_*λ,δ *_are close to those of *W*_*λ *_and the resulting estimates are nearly identical to those of the original IWLS (10). In this paper, we report  = 0 when  < 0.00005.

**Table 7:** The derivatives of the penalty functions.

Types	
LASSO	λ
SCAD	
HL	

### Other methods for sparse principal component analysis

[7] also exploit the regression property of PCA in order to obtain sparse loadings. They proposed an alternating minimization algorithm to minimize the criterion(11)

for deriving the first sparse loading vector *v*_1_. Given *θ*, this optimization problem becomes a naive elastic net problem for *v*_1_. Given *v*_1 _, *θ *can updated from SVD of *X*^*T*^*X v*_1_. These two steps are repeated until *v*_1 _converges. Following [25], (11) is different from our objective function (5) even when we use the same penalty function. In fact, (5) is very close to the objective function of [26], but we put the normalization constraint of the loading inside iterated procedure so that it could make a different result. In this paper, we used the function spca() in the R-package elasticnet for the EN method in the simulation studies.

### Condition-number constraint for SPCA

As shown in the previous examples, the SPCA approaches above may not produce sufficient sparsity. For the moment suppose *n *≥ *p*; the case where *n *<*p *can be dealt with by transposing the data; see the note below. From (2) we have the eigenvalue decomposition of the sample covariance matrix as

where Λ = diag(*l*_1_, ..., *l*_*p *_and  for *i *= 1, ..., *p *is the eigenvalues of *S*_*X *_in non-increasing order (*l_1 _≥ … ≥ l_*p *_≥ *0). Let the *p *× 1 random vectors *x*_1_, …, *x*_*n *_be rows of *X *that have zero mean vector and true covariance matrix Σ with the non-increasing eigenvalues, *λ*_1 _≥ ... ≥ *λ*_*p*_. When our goal is to estimate Σ, the sample covariance matrix *S*_*X *_can be used. Many applications require a covariance estimate that is not only invertible but also well-conditioned. An immediate problem arises when *n *<*p*, where the estimate *S*_*X *_is singular. Even when *n *>*p*, the eigen-structure tends to be systematically distorted unless *p*/*n *is small [27], resulting in ill-conditioned estimator for Σ.

[28] showed that the eigenvalues of *S*_*X *_are more dispersed than those of the true covariance matrix, i.e. *l*_1 _tends to be larger than *λ*_1 _and *l*_*p *_tends to be smaller than *λ*_*p*_. To overcome this difficulty, [29] proposed a constraint on the condition number to achieve a better covariance estimation. The optimization problem with the condition-number constraint can be formulated as(12)

where *A *≼ *B *denotes that *B *- *A *is positive semidefinite and *t >*0. Given *κ*_max_, for *t *[29] proposed to use

where *α *∈ {1, ... *p*} is the largest index such that 1/*l*_*α *_<t* and *β *∈ {1,..., *p*} is the smallest index such that 1/*l*_*β *_>*κ*_max_t*. Their covariance estimators are(13)

where the eigenvalues . To estimate the shrinkage parameter *κ*_max_, they proposed to use the *K*-fold cross validation.

From (2) and (13), we can reconstruct X^* ^with same singular vectors but shrunken singular values, i.e.(14)

where D* is *n *× *p *matrix with (*i*, *i*)th diagonal element . Thus, for condition-number constrained PCA we use X* instead of the original data matrix *X*. As the procedure yields extremely sparse loading vectors, we call it SSPCA, for super-sparse PCA.

[29] considered the estimation of covariance matrix when *p *is not very large. However, for large *p *such as over 10,000 in gene expression data, it becomes computationally too intensive. Because the aim is to obtain a few singular vectors, not whole *p *singular vectors, when *p *>*n *in this paper we propose to apply the above algorithm to *X*^*T *^and the results are transformed back appropriately.

### Modified NIPALS algorithm for SPCA and SSPCA

For SPCA we replace step 1 in the NIPALS algorithm by

where  is defined in (9). For SSPCA we also apply this modified step, but replace *X *by X* defined in (14).

### Tuning parameter selection

To complete the proposed algorithm we need to estimate the tuning parameters *θ *and *λ *= *ϕ*/*θ *in (9) and (10), respectively. First we note that from (7), marginally, *v*_1 _has mean zero and variance *θ*, so we use , where  is the estimated first loading vector from ordinary PCA and  is the sample mean of . We use *K*-fold cross-validation for λ. Following [30], we select λ which maximizes the test sample variance

where  is the estimated loadings from the *k*th training sets (the whole data without the *k*th validation set) and *S*_*X*[*k*] _is the sample variance based on the *k*th validation set. For the numerical studies in Section we use *K *= 5.

## Authors' contributions

The first two authors (DHL and WJL) contributed equally to this work. YJL and YP conceived the study and wrote the manuscript, DHL and WJL performed data analysis and wrote the manuscript. All authors read and approved the final manuscript.

## Supplementary Material

Additional file 1 The supplementary report documents details on plot of the SSPCA scores, and Gene Ontology analysis of ordinary PCA. Click here for file
