# Supplementary material for: Super-sparse principal component analyses for high-throughput genomic data
Source: BMC Bioinformatics. 2010 Jun 2;11:296. doi: 10.1186/1471-2105-11-296 (PMC2902448; doi:10.1186/1471-2105-11-296)
Supplement: Additional file 1 — The supplementary report documents details on plot of the SSPCA scores, and Gene Ontology analysis of ordinary PCA. [file 1471-2105-11-296-S1.PDF]

Additional File

Super-sparse principal component analyses for high-throughput genomic data

Donghwan Lee, Woojoo Lee, Youngjo Lee and Yudi Pawitan

Department of Statistics, Seoul National University, South Korea

Department of Medical Epidemiology and Biostatistics,

Karolinska Institutet, Stockholm, Sweden.

May 28, 2010

Figure 1 shows the normal quantile plots of the loading vectors from the five different methods. The sparsity requirements of the SPCA methods (either the Lasso or HL penalties) or even the SSPCA with Lasso penalty are not strong enough to yield substantially distinct results from the ordinary PCA. The strong restriction using the SSPCA with HL penalty can deliver such results.

Plot of the SSPCA scores

Figure 2 shows the scatterplot matrix of the first 3 SSPCA scores. The symbols 1 to 9 refer to the following cancer tissues respectively: 1=breast, 2=central nervous system, 3=colorectal, 4=lung, 5=leukemia, 6=melanoma, 7=ovarian, 8=prostate, 9=renal. From the plot of the first vs the second scores, the largest contrasts from the extreme corners occur between central nervous system (2), colorectal (3) and leukemia (5). This means that the biological processes that are enriched – as shown in the main paper – are likely to vary significantly between these cancers. Intriguingly, the breast cancers (1) are spread across the other cancer types. This might indicate that, at RNA level, the breast cancer has more diverse underlying biological processes than other cancers.

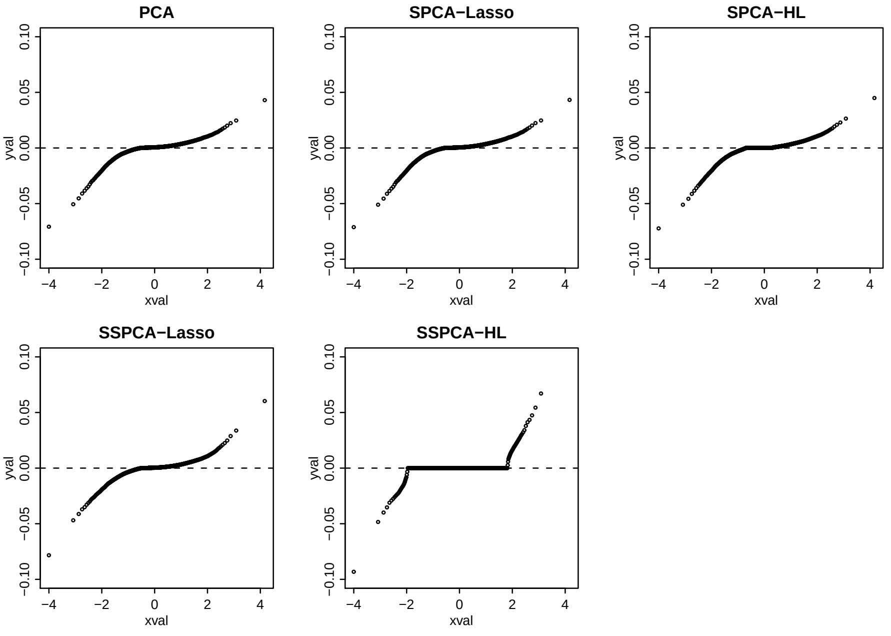

Figure 1: The normal quantile plots of the first loading vectors from the 5 different methods.

Gene Ontology analyses of the ordinary PCA

For comparisons we provide here the gene ontology (GO) analyses of the ordinary PCA results. We limit to the first 3 PCA. Since it is not obvious how many non-zero loading values to use, we simply take 1000 top-ranking genes in absolute values, which are approximately the same number as the number of non-zero loadings using SSPCA.

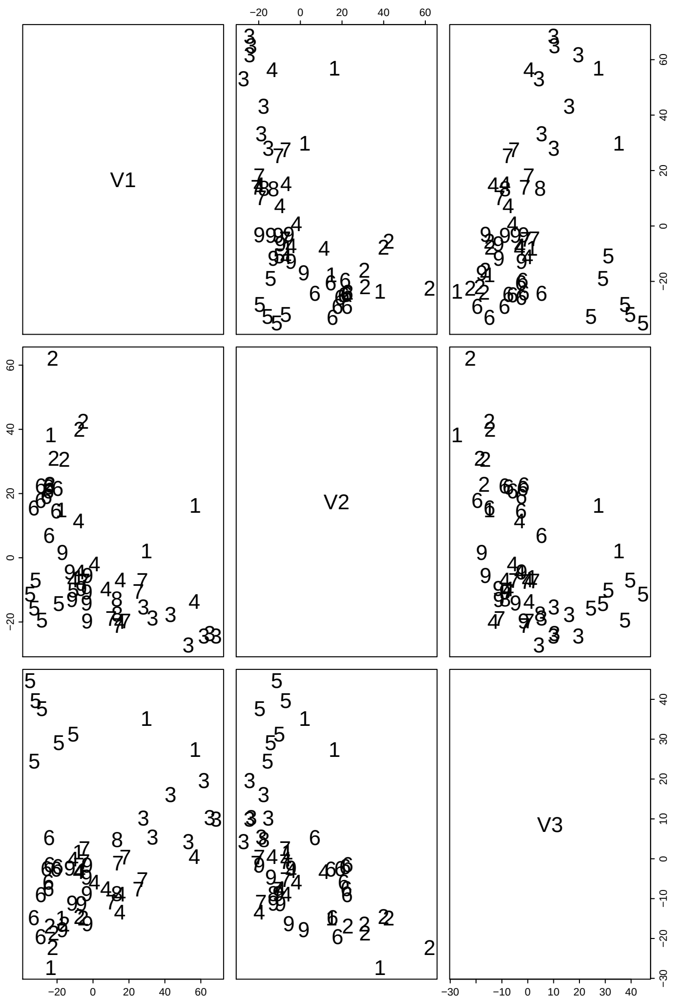

Figure 2: The scatterplot matrix of the first 3 SSPCA scores.

GO ID	GO Term	P-value(1)	P-value(2)	P-value(3)
1 GO:0051272	positive regulation of cell motion	3.1e-08		
2 GO:0030335	positive regulation of cell migration	3.5e-08		
3 GO:0030198	extracellular matrix organization	5.0e-08		0.00031
4 GO:0030199	collagen fibril organization	1.4e-07		
5 GO:0001666	response to hypoxia	5.5e-07		
6 GO:0032101	regulation of response to exterl stimulus	1.2e-06		
7 GO:0050900	leukocyte migration	2.9e-06		
8 GO:0043062	extracellular structure organization	6.2e-06		0.00024
9 GO:0051271	negative regulation of cell motion	6.5e-06		
10 GO:0007596	blood coagulation	1.6e-05		
11 GO:0050817	coagulation	1.6e-05		
12 GO:0033627	cell adhesion mediated by integrin	1.8e-05		
13 GO:0033631	cell-cell adhesion mediated by integrin	1.8e-05		
14 GO:0043542	endothelial cell migration	3.1e-05		
15 GO:0007599	hemostasis	4.4e-05		
16 GO:0007050	cell cycle arrest	4.8e-05		
17 GO:0050818	regulation of coagulation	5.1e-05		
18 GO:0031589	cell-substrate adhesion	5.5e-05		
19 GO:0007229	integrin-mediated sigling pathway	5.6e-05		0.00016
20 GO:0050819	negative regulation of coagulation	5.7e-05		
...				
66 GO:0002009	morphogenesis of an epithelium	9e-04		
67 GO:0048545	response to steroid hormone stimulus	0.00097	0.00047	
68 GO:0048066			4.2e-10	
69 GO:0043473			1.5e-09	
70 GO:0002504			2.3e-06	
71 GO:0030318			4.8e-06	
72 GO:0050931			8.6e-06	
73 GO:0006583			1.7e-05	
74 GO:0048069			1.7e-05	
75 GO:0043627	response to estrogen stimulus		3e-05	
76 GO:0042438			4.9e-05	
77 GO:0006582			0.00011	
78 GO:0006726			0.00032	
79 GO:0042441			0.00032	
80 GO:0019882			0.00041	
81 GO:0030155	regulation of cell adhesion		0.00071	
82 GO:0022614	membrane to membrane docking		0.00076	
83 GO:0009072			0.00081	
84 GO:0006570			0.00091	
85 GO:0006805				0.00035
86 GO:0006081				0.00057
87 GO:0009410				0.00061
88 GO:0002523	leukocyte migr. during inflam. resp.			0.00064

Table 1: The 88 most enriched biological process GO terms (set to have P-values< 0.001) and the associated P-values for the first 3 principal components from ordinary PCA. The numbers of nonzero loadings are set to be equal to those from SSPCA, which are 1,260, 681 and 375 for the first 3 principal components
